# Mast cells and mast cell tryptase enhance migration of human lung fibroblasts through protease-activated receptor 2

**DOI:** 10.1186/s12964-018-0269-3

**Published:** 2018-09-15

**Authors:** Mariam Bagher, Anna-Karin Larsson-Callerfelt, Oskar Rosmark, Oskar Hallgren, Leif Bjermer, Gunilla Westergren-Thorsson

**Affiliations:** 1 Unit of Lung Biology, Department of Experimental Medical Sciences, Lund University, BMC C12, 221 84 Lund, Sweden; 2 Department of Respiratory Medicine and Allergology, Skåne University Hospital, Lund University, Lund, Sweden

**Keywords:** Human lung fibroblast, Lung, Mast cell, Migration, Protease activated receptor 2, Tryptase

## Abstract

**Background:**

Mast cells may activate fibroblasts and contribute to remodeling processes in the lung. However, the mechanism behind these actions needs to be further investigated. Fibroblasts are major regulators of on-going remodeling processes. Protease activated receptor 2 (PAR2) expressed by fibroblasts may be activated by serine proteases, such as the mast cell mediator tryptase. The objective in this study was to investigate the effects of mast cells and specifically mast cell tryptase on fibroblast migration and the role of PAR2 activation.

**Methods:**

Human lung fibroblasts (HFL-1) were cultured together with human peripheral blood-derived mast cells or LAD2 mast cells and stimulated with either conditioned medium from LAD2 cells or tryptase. Analyses of immunological stimulation of mast cells by IgE/anti IgE in the co-culture system were also performed. The importance of PAR2 activation by mast cells and mast cell tryptase for the migratory effects of fibroblasts was investigated by pre-treatment with the PAR2 antagonist P2pal-18S. The expression of PAR2 was analyzed on fibroblasts and mast cells.

**Results:**

The migratory capacity of HFL-1 cells was enhanced by blood-derived mast cells (*p* < 0.02), LAD2 cells (*p* < 0.001), conditioned medium (*p* < 0.05) and tryptase (*p* < 0.006). P2pal-18S decreased the induced migration caused by mast cells (*p* < 0.001) and tryptase (*p* < 0.001) and the expression of PAR2 was verified in HFL-1 cells. Mast cells immunologically stimulated with IgE/Anti IgE had no further effects on fibroblast migration.

**Conclusions:**

Mast cells and the mast cell mediator tryptase may have crucial roles in inducing lung fibroblast migration via PAR-2 activation, which may contribute to remodeling processes in chronic lung diseases.

**Electronic supplementary material:**

The online version of this article (10.1186/s12964-018-0269-3) contains supplementary material, which is available to authorized users.

## Background

Mast cells (MC) are involved in the innate immune response and play a major role in allergic diseases by releasing pro-inflammatory mediators such as histamine, prostaglandins and proteases such as tryptase and chymase [1]. During recent years, it has been suggested that mast cells may also have an important role in non-allergic chronic lung diseases, including chronic obstructive pulmonary disease (COPD) [2], asthma [3] and idiopathic pulmonary fibrosis (IPF) [4, 5]. There are two major subtypes of human mast cells; mucosal mast cells with granules containing tryptase (MC_T_) and connective tissue mast cells with granules containing both chymase and tryptase (MC_TC_). Interestingly, the MC_TC_ have been reported to increase at areas of inflammation and fibrosis [6]. Previous studies have shown increased numbers of mast cells in remodeled lung tissue, especially in fibrotic lesions [7] that correlated with the synthesis of type I collagen and other extracellular matrix (ECM) proteins [8]. Fibroblasts are mesenchymal cells that are crucial for maintaining ECM homeostasis in the lung [1, 9]. Myofibroblasts have morphological features of both fibroblasts and smooth muscle cells. These cells are increased in number in chronic lung diseases and have been suggested to contribute to tissue remodeling processes [10]. Previous studies imply that mast cell mediators are involved in fibroblast differentiation into myofibroblasts [11]. Mast cell mediators, such as tryptase, may induce ECM synthesis, migration and proliferation in fibroblasts, resulting in airway remodeling. Mast cell tryptase has been suggested to be an important factor driving abnormal remodeling in chronic lung diseases by stimulating fibroblasts either directly, or by growth factor induction [12–14]. Previous studies suggest that mast cell tryptase may induce mitogenic activity in fibroblasts [13, 14], as well as increase the production of type I pro-collagen [8]. PAR2 is a G-protein coupled receptor activated by proteolytic cleavage by serine proteases, including tryptase [15]. The specific cleavage of the amino-terminus of PAR2 by tryptase, exposes a new amino-terminus, which interacts with another part of the cleaved receptor. This leads to the activation of downstream cell signaling pathways, involving binding to β-arrestins and activating ERK1, 2 [16]. PAR2 is expressed by several cell types and may be involved in tissue remodeling by inducing fibroblast migration, differentiation and ECM production [17, 18]. The expression of PAR2 has been reported to be elevated in fibroblasts and myofibroblasts in IPF [19], as well as in inflammatory diseases [20]. Despite evidence that mast cells activate fibroblasts and contribute to remodeling, the mechanism for this interaction is poorly understood. For this reason, this study was designed to test the hypothesis that mast cells influence the migratory capacity of fibroblasts through PAR2.

## Methods

### Mast cells

Human peripheral blood derived mast cells (PBdMC) were generated by separating and isolating peripheral blood mononuclear cells. All blood donors gave their written informed consent to participate in the study, which was approved by the regional Ethics Committee in Lund (KIT 2010–29). The peripheral blood mononuclear cells were separated by Ficoll-plaque (Sigma Aldrich, St Louis, MO, US) [21] density gradient centrifugation and progenitor cells were separated using anti-CD34 magnetic beads with FcR blocking reagent (Miltenyi Biotec, Bergisch Gladbach, Germany). The progenitor cells were incubated (5% CO_2_, 37 °C) in StemPro®-34 Serum-free medium with Stem Pro-34 nutrient supplement (Invitrogen, Waltham, Massachusetts, USA), 1% Penicillin-Streptomycin (PEST), 1% L-glutamine (both from Gibco BRL, Paisley, UK), and the cytokines IL-3 (30 ng/ml, just first week), IL-6 (100 ng/ml, continuously from first week) and SCF (100 ng/ml, continuously from first week) (Stem cell factor) (all from Peprotech, Stockholm, Sweden). The progenitor cells were incubated for 6 weeks in order to differentiate into mature mast cells (PBdMC), as a mix of both MC_TC_ and MC_T_ [22, 23]. The cell medium was changed by weekly hemidepletion. The mast cells were used within 10 weeks after the first day of isolation from peripheral blood [24–26]. The LAD2 mast cells (Dr. Arnold Kirshenbaum, Laboratory of Allergic Diseases, NIAID, Bethesda, US) were cultured in 500 mL StemPro 34 medium supplemented with 13 mL StemPro nutrients, 1% PEST, 1% glutamine and 100 ng/ml SCF. The cell concentration was doubled within 10 to 14 days and Stempro 34 medium mixture was added to the LAD2 cell culture by weekly hemidepletion, with a target cell density around 0.3–0.5 × 10^6^ cells/ml. A study described that LAD2 mast cells express both tryptase and chymase, however, in a lower concentration than primary skin mast cells [27].

### Characterization of peripheral blood derived mast cells (PBdMC)

#### Transmission electron microscopy analysis

PBdMC were fixed with 4% formaldehyde and placed on 200-mesh, thin-bar copper grids. Uranyl acetate and lead citrate were added to stain the grids for imaging with transmission electron microscopy (CM-10 TEM microscope, Philips, Eindhoven, Netherlands) [28].

#### Fluorescence-activated cell sorting (FACS) of PBdMC

Differentiated PBdMC were harvested, washed and non-specific binding was blocked. The cells were stained with the following fluorophore conjugated antibodies: CD34 (cat.no 555824), CD117 (cat.no 550412), CD88 (cat.no 550494, all from BD Biosciences Pharmingen, Franklin Lakes, NJ, USA). 7-amino-actinomycin D (Sigma Aldrich, St Louis, MO, US) was used for excluding dead cells. Samples were acquired on a FACSCalibur™ cell analyzer and analysis was performed using the CellQuest software (both from BD Bioscience). Results are presented as per cent positively stained cells as compared to isotype controls.

#### Immunocytochemistry staining of mast cells and human lung fibroblasts

Human fetal lung fibroblasts (HFL-1; ATCC, Rockville, USA) were used between passages 17 and 20. HFL-1 (10000 cells/well) and co-cultures of HFL-1 (10,000 cells/well) and LAD2 cells (7500 cells/well), were seeded in 4-well glass chamber slides (154526; Thermo Scientific, Waltham, MA) and incubated overnight at 37 °C, 5% CO_2_. The cells were then fixed in 4% paraformaldehyde for 15 min and blocked in 2% BSA-TBS containing 5% goat serum (Vector laboratories, Burlingame, CA) and 0.2% Triton-X for 30 min, followed by washing twice in tris-buffered saline (TBS). Cells were incubated for 60 min with monoclonal tryptase antibody (M7052, Dako, Glostrup, Denmark) and monoclonal PAR2 antibody (cat.nr: 35–2300, Thermo Fisher Scientific, Waltham, Massachusetts, USA). The cells were then washed in TBS and incubated for 45 min with secondary antibodies (Thermo Fisher Scientific), goat anti-mouse IgG2a (Alexa Fluor® 647, A21241), goat anti-mouse IgG1 (Alexa Fluor® 647, A21240) or goat anti-mouse IgG1 (Alexa Fluor® 555, A21127), followed by washing in TBS. Nuclei were stained by using DAPI containing mounting medium (Dako). Cells were imaged using a VS120 slide scanner with XV image processor L100 VS-ASW (Olympus, Tokyo, Japan). Image viewer software VS-OlyVIA (version 2.9) (Olympus Soft Imaging solutions GmbH; Münster, Germany) was used for image visualization.

### Mast cell degranulation by β-hexosaminidase

PBdMC and LAD2 cells were sensitized with IgE from human myeloma plasma (100 ng/mL, Calbiochem, Merck Chemicals, Beeston, Nottingham, UK) and incubated overnight in humidified 5% CO_2_, 37 °C. After sensitization, the cells were degranulated by stimulation with anti-human IgE antibody (0.5 μg/mL, KPL, Gaithersburg, MD, USA), and washed three times with HEPES buffer. The cells were resuspended in HEPES buffer and 5000–10 000 cells/well were plated in a 96-well plate. A calcium ionophore (A23187, Sigma Aldrich) was used as a positive control. The β-hexosaminidase assay was performed according to published protocol [29, 30]. According to the protocol, mast cell degranulation by IgE/Anti IgE occurs seconds after stimulation and reaches its peak after 25 min. After that, the β-hexosaminidase release becomes constant. The time point 40 min was therefore chosen to assure that maximum degranulation and mediator release was obtained in the test system.

### Cell proliferation

In vitro cell proliferation assays were performed as previously described [31, 32]. The HFL-1 cell cultures were grown in Dulbecco’s Modified Eagle Medium (DMEM) culture medium (DMEM, Sigma-Aldrich, St Louis, MO) supplemented with 10% fetal clone serum (FCIII, Thermo Scientific, Waltham, MA), 1% PEST and 1% L-glutamine. HFL-1 (6,500 cells/well; 200 μl/well) respectively co-cultures (200 μl/well) with HFL-1, (6,500 cells/well) and mast cells (LAD2, 5,000 cells/well) were seeded in 6 replicates in four 96-well plates. The cells were incubated for 6 h in 37 °C with 5% CO_2_ in order to allow the cells to attach. Afterwards, the cells were washed with PBS, and new DMEM medium containing 10% respectively 0.4% FCIII was added to the fibroblasts and the co-cultures in the presence or absence of mast cell tryptase (75 ng/ml, Sigma Aldrich). Conditioned medium from LAD2 cells was obtained in medium containing 10%, respectively 0.4% FCIII serum (0.1 × 10^6^ cell/200 μl) [31, 32].

The cells in each plate were stimulated with the same treatment and allowed to grow up to 72 h after stimulation. After each time point, the medium was aspirated and cells were washed with PBS and fixed in glutaraldehyde. The cell density was optimized by using a cell nucleus staining solution, crystal violet (0.1%) and incubated for 30 min. After several washing steps in order to remove the unbound dye, the absorbed crystal violet in the cell nucleus was dissolved by adding Triton-X (1%) and incubated over night at 4 °C. The absorbance, at 600 nm, was measured using a plate reader. The absorbance of the dissolved crystal violet is directly proportional to the cell density [31, 32].

### Cell migration

A scratch assay was used to investigate cell migration in vitro [33, 34]. HFL-1 cells from passage 15–19, with a cell density of 0.13 × 10^6^ cells/well (2 mL) were seeded in 6-well plates. For the co-culture, 0.1 × 10^6^ cells/well mast cells (PBdMC respectively LAD2) were re-suspended in DMEM medium (10% FCIII), and mixed together with fibroblasts (0.13 × 10^6^ /well) and seeded in 6-well plates, 2 mL/well. The cells were incubated in 37 °C with 5% CO_2_ for 72 h to become confluent. After starvation for 24 h in medium containing 0.4% FCIII, the cell monolayer was scratched as a cross from one edge to the other in each well using a 1 ml pipette tip. The cells were gently washed twice with PBS. DMEM with 0.4% FCIII was added with and without different stimulating factors as described below, and incubated in 37 °C with 5% CO_2_ for another 72 h. Images of the scratch were captured at 0, 24 h, 48 h and 72 h after the scratch using a TMS microscope (Nikon, Tokyo, Japan). The migratory capacity of HFL-1 cells was measured at 24, 48, 72 h as the percentage of cell-occupied space compared to time (0 h, the starting point when the scratch was made). A scratch was created on a cell monolayer and the movements of the cells, the cell migration, was observed during different time points. Images of each scratch captured after 24 h, 48 h, and 72 h was compared to the image of itself at time point 0, which was set as the reference point. A migration of 0% means the time point 0 h, where the scratch was made and the cells had not started their migration, the area of this scratch is set as the reference point, while a 100% migration means that the scratch (cross) has reached total closure. The remaining cell free area was inversely correlated with the ability of the cells to migrate, the less empty cell free area in the scratch the more have the cells migrated. The scratch area was measured with the TScratch software [34, 35], the central dot was used as reference point to locate where photos were taken. In order to perform experiments without being limited by PBdMC amounts, LAD2 cells were used in the following experiments.

#### Stimulation with mast cell tryptase

Fibroblasts in monoculture were stimulated with three different concentrations of mast cell tryptase (10 ng/ml, 50 ng/ml and 75 ng/ml).

#### Stimulation with IgE degranulated mast cells

The cells were sensitized by adding human IgE (100 ng/ml) at the same day as the starvation and then incubated overnight. Then human Anti IgE (0.5 μg/ml) was added to IgE sensitized mast cells to study the effect of immunological stimulation on fibroblast migration.

#### Stimulation with conditioned media from LAD2 cells and IgE degranulated LAD2 cells

The IgE sensitized and the non-sensitized LAD2 cells were resuspended in DMEM medium (0.4% FCIII). A cell density of 0.1 × 10^6^ cells/well (2 ml) was used also for the conditioned medium. Human Anti IgE (0.5 μg/ml) was added to the LAD2 cells with and without the IgE sensitization, and incubated for 1 h in 37 °C with 5% CO_2_. The cell-suspension was centrifuged and the cell supernatant was considered as the conditioned medium.

#### Pharmacological interventions with PAR2 antagonist

The pepducin lipopeptide PAR2 antagonist P2pal-18S (palmitate-RSSAMDENSE KKRKSAIK-NH2, GL Biochem, Shanghai, China) was used to investigate if PAR2 is involved in the pro-migratory effects. Fibroblasts were pre-treated with different concentrations (1, 10, 100 μM) of P2pal-18S for 1 h before stimulation with mast cell tryptase or addition of mast cells in the migration experiments.

### Gene expression, analysis for proteins and ELISA

For the gene expression analysis, HFL-1 and LAD2 cells were washed with PBS, and total RNA was isolated with RNeasy mini kit (Qiagen, GmBH, Hilden, Germany). The cell mRNA concentrations were measured using Nanodrop ND-100 (Nano Drop Technologies, Delaware, Maryland, USA). Superscript II (Invitrogen) was added for the reverse-transcription of mRNA, followed by adding the PAR2 and F2RL-1 primers to the cDNA. Afterwards, CYBR Green Master Mix (Thermo Scientific) was added and reverse transcription PCR was performed using a StepOnePlus Real Time PCR system (Applied Biosystems, Waltham, Massachusetts, USA). The housekeeping gene 18S was used as reference gene and quantifications were performed according to the manufacturer’s instructions. Four different time-points were investigated; 0 h, 24 h, 48 h and 72 h, according to the cell migration studies. Cell medium and lysate from each well were collected and stored at − 20°C for further investigations. The release of tryptase into the medium was quantified by ELISA and performed according to the manufacturers’ instructions (Tryptase/TPSAB1, B2 PicoKine ELISA Kit, Nordic Diagnostica, Billdal, Sweden).

### Data analysis, calculations and statistical methods

**Fig. 1:**
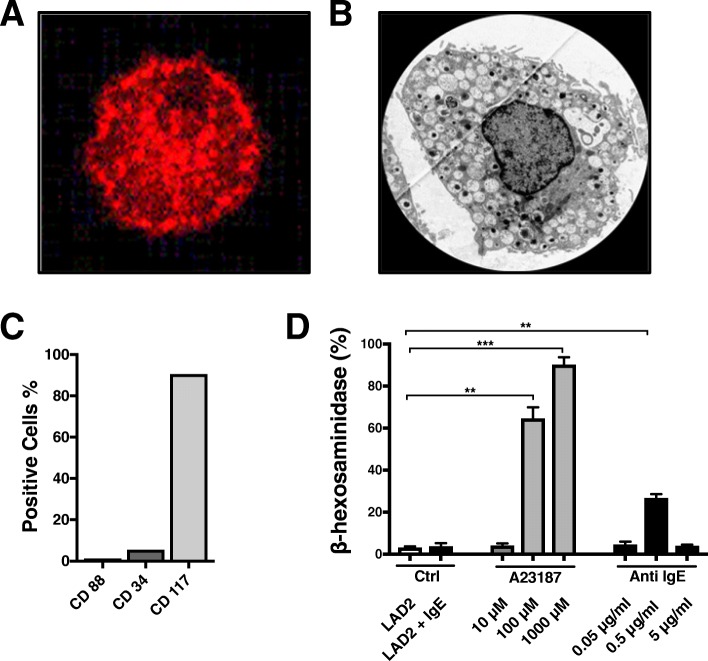
Characterization of peripheral blood-derived mast cells (PBdMC) and LAD2 cells. Immunofluorescence staining for tryptase in mast cells, representative staining is shown in red for tryptase (**a**). Transmission electron microscopy image of a mature PBdMC (**b**). Flow cytometry of PBdMC using antibodies against CD117: Mast cell marker (c-Kit), CD34: Progenitor cell marker, CD88: Marker for some mast cells (c5a-receptor) (**c**). Mast cell degranulation was measured by the ability to release β-hexosaminidase. The calcium ionophore A23187 efficiently degranulated LAD2 cells in a concentration-dependent manner, IgE/Anti IgE stimulation also triggered degranulation of LAD2 cells but at a lower level than A23187 (**d**). The maximum effect was observed at 0.5 μg/ml of Anti IgE. (mean ± SD, 3 individual experiments with 3 technical replicates in each experiment, *p < 0.05, **p < 0.01 and ***p < 0.001)

**Fig. 2:**
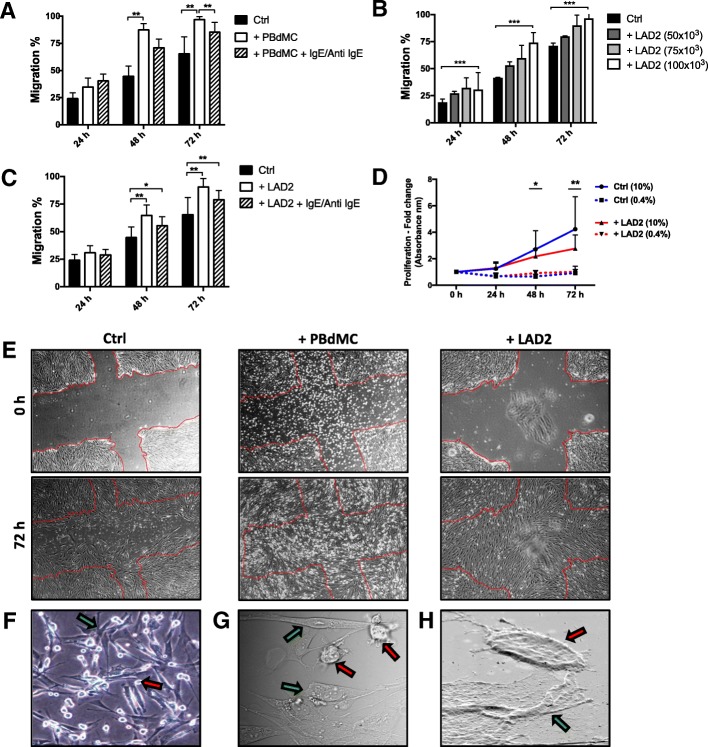
Effect of mast cells on migratory capacity and proliferation of human lung fibroblasts, HFL-1. PBdMC enhanced the migratory capacity of HFL-1. Anti IgE stimulated PBdMC had a decreased pro-migratory effect compared to non-stimulated PBdMC (**a**). LAD2 cells, similarly to PBdMC, enhanced the migratory capacity of fibroblasts in a concentration-dependent manner, where the highest effect could be observed at 1 × 10^5^ of LAD2 cells (**b**). There was no difference on the migration of HFL-1 in a co-culture with Anti IgE stimulated LAD2 compared to the non-stimulated LAD2 (**c**). The migratory capacity of HFL-1 cells was measured at 24, 48 and 72 h as the percentage of cell-occupied space compared to time 0 h when the scratch was made. LAD2 decreased proliferation of HFL-1 in co-cultures at 10% serum concentration, and did not have any effect on proliferation of HFL-1 at 0.4% serum concentration (**d**). The proliferation of HFL-1 was measured at 24, 48 72 h. The cell growth was determined using time-point 0 h as the reference point. (mean ± SD, *n* = 3 individual experiments with 4–6 technical replicates). Representative images of HFL-1 cells at 0 h (upper panels) and 72 h (lower panels). Images show 0.4% medium control, co-culture with PBdMC and co-culture with LAD2 (**e**) (mean ± SD, *n* = 3 individual experiments with 2 technical replicates in each experiment, *p < 0.05, **p < 0.01 and ***p < 0.001). The image of co-culture with PBdMC at time point 0 h, show recently added mast cells floating in the cell medium before attached to HFL-1 (**e**). Light microscopy images of co-cultures of HFL-1 (green arrow) and PBdMC (red arrow) (**f**), confocal microscopy images of HFL-1 and LAD2 co-cultures (**g**), Scanning electron microscopy (SEM) images HFL-1 and LAD2 (**h**)

**Fig. 3:**
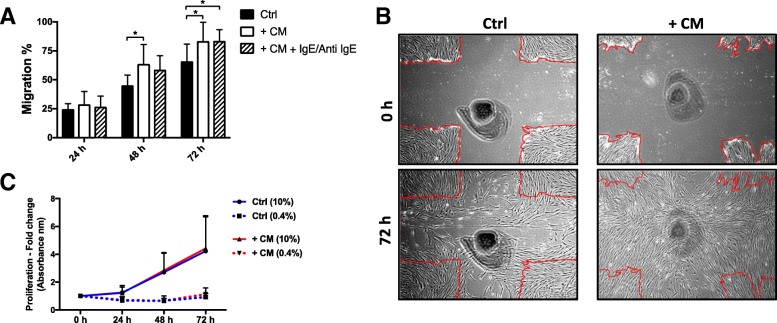
Effect of mast cell degranulation on HFL-1 migration. Conditioned medium from LAD2 cells enhanced the migration capacity of HFL-1 (**a**). However, there was no difference in migration between HFL-1 cells that were treated with conditioned medium from IgE/Anti IgE stimulated LAD2 cells, compared to non-stimulated cells (**a**). Representative images of HFL-1 cells from starting point at 0 h (upper panels) and after 72 h (lower panels), HFL-1 controls and fibroblasts stimulated with conditioned medium from LAD2 cells (**b**). The migratory capacity of HFL-1 cells was measured at 24, 48, 72 h as the percentage of cell-occupied space compared to time = 0 h when the scratch was made (mean ± SD, *n* = 3 individual experiments with 2 technical replicates in each experiment). Conditioned medium did not have any proliferative effects on HFL-1 neither in 0.4% nor 10% serum concentration (**c**). Proliferation of HFL-1 was measured at 24, 48 and 72 h after stimulation (mean ± SD, *n* = 3 individual experiments with 4–6 technical replicates in each experiment, *p < 0.05, **p < 0.01 and ***p < 0.001)

**Fig. 4:**
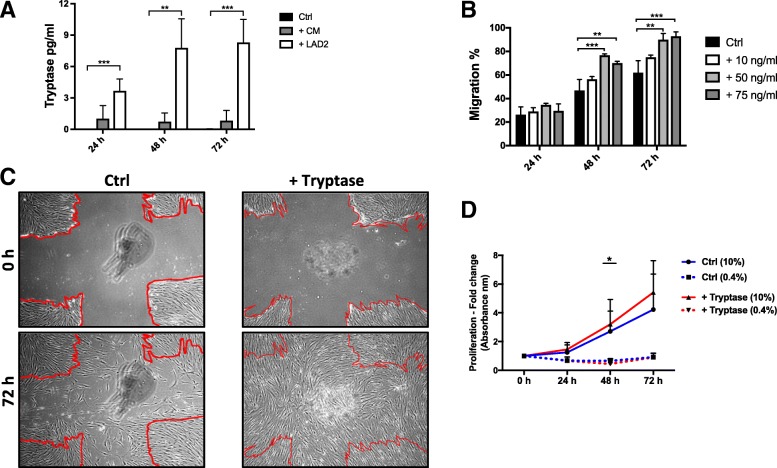
The effect of mast cell tryptase on the migration capacity of HFL-1 cells. Co-cultures with LAD2 and HFL-1 showed an increase in tryptase concentration over time, while conditioned medium had unchanged tryptase concentrations over time. HFL-1 did not show any release of tryptase (**a**). Tryptase enhanced the migration capacity of HFL-1 at 50 ng/ml and 75 ng/ml, (**b**). Representative images of migration from starting point 0 h (upper panels) and after 72 h (lower panels) in control wells with only HFL-1 fibroblasts and after stimulation with tryptase (**c**). The migratory capacity of HFL-1 cells was measured at 24, 48 and 72 h as the percentage of cell-occupied space compared to time = 0 h when the scratch was made. (mean ± SD, *n* = 3 individual experiments with 2 technical replicates in each experiment). Tryptase (75 ng/ml) increased the proliferative rate of HFL-1 when cultured in 10% (*p* < 0.03 at 48 h) serum, but did not have any effects on HFL-1 proliferation in 0.4% serum (**d**) (mean ± SD, *n* = 3 individual experiments with 4–6 technical replicates in each experiment, *p < 0.05, **p < 0.01 and ***p < 0.001)

**Fig. 5:**
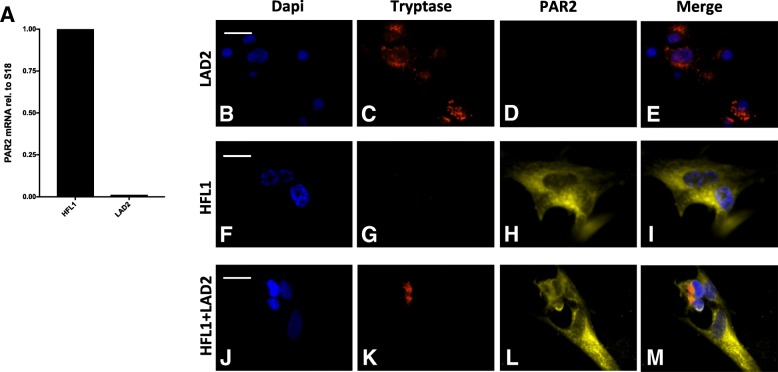
Expression of PAR2 in HFL-1 cells. RTqPCR was used to confirm the expression of the serine protease receptor PAR2 on HFL-1. The relative gene expression was related to the housekeeping gene 18 s (*n* = 2, 1 experiment). Human lung fibroblasts, HFL-1, showed a high gene expression for PAR2, while LAD2 cells did not show any PAR2 gene expression (**a**). Expression of PAR2 examined in monolayer cultures of HFL-1, LAD2, and in co-culture of HFL-1 and LAD2. Representative images are shown with PAR2 staining in yellow, mast cell specific tryptase-staining in red and nuclei stained with DAPI in blue (**b-m**). LAD2 cells stained by PAR2 antibodies showed no positive receptor staining (**d**), while HFL-1 fibroblasts showed a positive expression (**h**). HFL-1 showed no positive staining for tryptase (**g**), while mast cells were immunopositive (**c**). Representative scale bars are shown at the beginning of each figure (**b**, **f**, **j**) and indicated as 20 μm

**Fig. 6:**
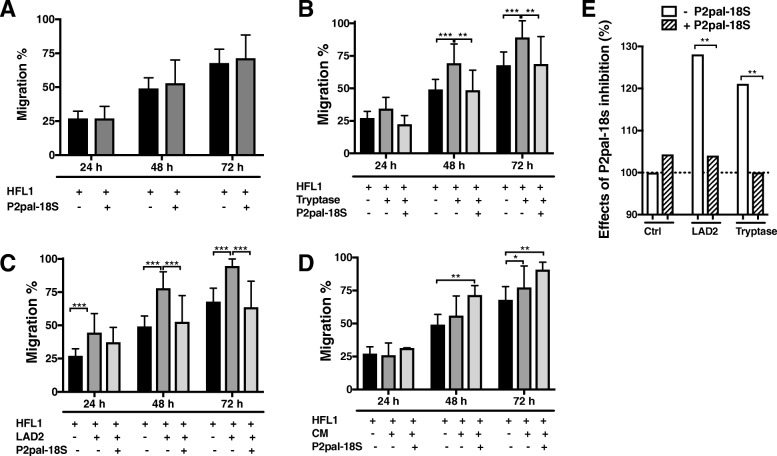
Effect of PAR2 antagonist on migratory capacity of HFL-1. The PAR2 antagonist P2pal-18S (10 μM), did not have any significant effect on the migratory capacity of the HFL-1 (**a**). P2pal-18S inhibited the pro-migratory effect of tryptase at 48 h and 72 h (**b**), and in co-cultures of fibroblasts and LAD2 cells at 48 h and 72 h (**c**). Conditioned medium (CM) increased the fibroblast migration, in presence (48 h and 72 h) and absence (72 h) of stimulation with P2pal-18S (**d**). The enhanced migration of HFL-1 caused by LAD2 decreased from 28 to 4% at 48 h, after stimulation with PAR2 antagonist, P2pal-18S. Tryptase enhanced the HFL-1 migration by 21%, while this effect was reduced to 0.015% at 48 h, after stimulation with P2pal-18S (**e**). The migratory capacity of HFL-1 cells was measured at 24, 48 and 72 h as the percentage of cell-occupied space compared to time point 0 h. The statistical analysis was performed using linear mixed models (mean ± SD, *n* = 3–4 individual experiments with 2–3 technical replicates in each experiment, *p < 0.05, **p < 0.01 and ***p < 0.001)

Statistical analyses and graphs were generated using the GraphPad software (GraphPad Software Prism 7, La Jolla, USA). For two-group comparisons in Figs. 1, 2, 3, 4 and 5, Student’s t-test were used. To investigate migration over time and in response to stimulations and inhibitors, linear mixed models were used in Fig. 6 and in Additional file 1: Figure S1. To investigate the effect of time and different cell types in the area of cell migration, linear mixed models were employed. These types of models were chosen in part to account for the repeated measurement nature of the experimental design and in part due to its ability to handle the unbalanced design, i.e. that the number of observations for each cell type differs within one experiment. In order to investigate whether cell migration was different among the different cell stimulations over time, an interaction term of cell stimulations and time was included in the mixed model analysis. If the interaction term was not significant, results from a model without the interaction term is presented. These data analyses were performed using SPSS version 22 (SPSS, Inc., Chicago IL). *p*-values of *p < 0.05, **p < 0.01 and ***p < 0.001 were considered as statistically significant.

## Results

### Characterization of peripherally blood derived mast cells

Matured peripherally blood derived mast cells (PBdMC) were differentiated from isolated human blood progenitor cells. Different characterization methods were used to confirm the mast cell phenotype. PBdMC were positive for tryptase, which is the main mast cell protease released during degranulation [30] (Fig. 1a). Transmission electron microscopy (TEM) showed mature PBdMC containing secretory vesicles (granules) (Fig. 1b), characteristic for mast cells. Although, many of the granules were empty indicating that the cells probably had degranulated (Fig. 1b). The PBdMC were positive for CD117 (c-KIT). As expected, PBdMC were negative for the progenitor cell marker CD34, a marker that is gradually lost during the differentiation process [26, 36]. The PBdMC were negative for CD88, a marker that can be expressed by some mast cells (Fig. 1c). The efficiency of mast cell degranulation was analyzed by β-hexosaminidase. Degranulation of mast cells occurred after IgE/Anti IgE stimulation, where 0.5 μg/ml (*p* < 0.004) was the most potent concentration, confirming the immunological degranulation being effective. The calcium ionophore A23187 was used as a positive control (Fig. 1d).

### Effects of mast cells on migration and proliferation of fibroblasts

PBdMC significantly enhanced the migration of human lung fibroblasts compared to controls at both 48 h (*p* < 0.003) and 72 h (*p* < 0.02) (Fig. 2a, e). PBdMC cells were stimulated with IgE/Anti IgE in order to examine the role of an induced immunological degranulation on fibroblast migration. Anti IgE stimulation reduced the migratory effect of PBdMC after 48 h (*p* < 0.003, Fig. 2a). Due to limitations in PBdMC amounts, LAD2 cells were instead used in the following experiments. Different cell concentrations of LAD2 cells showed a concentration-dependent effect on the migration of HFL-1, where the highest effect could be observed at 0.1 × 10^6^ LAD2 cells/ 0.13 × 10^6^ HFL-1 (Fig. 2b). LAD2 cells significantly enhanced the migration capacity of HFL-1 at 48 h (*p* < 0.0001) and 72 h (*p* < 0.00005) (Fig. 2c, e), similarly to the effect of PBdMC. The pro-migratory effect on fibroblasts was however not affected by IgE/Anti IgE stimulation of the LAD2 cells (Fig. 2c). To investigate whether the observed effect of mast cells on fibroblasts also affected proliferation, we examined proliferation in medium containing 10% and 0.4% serum. The lower serum concentration (0.4%) was the same as in the migration experiments, while the higher (10%) was used as a positive control. In 10% serum, LAD2 decreased proliferation of HFL-1 at the time-points 48 h (*p* < 0.013), and 72 h (*p* < 0.009), whereas there was no significant effect of mast cells on the proliferation rate of HFL-1 in 0.4% serum (Fig. 2d). Images of co-cultures with fibroblasts and mast cells clearly indicated a close cell-cell interaction between these cells in vitro; (Fig. 2f–h).

### Effect of conditioned medium on fibroblasts

In order to examine the effects of soluble factors released by mast cells, conditioned medium (CM) from LAD2 mast cells was added to the fibroblasts and the effect on migration was observed. CM enhanced the migratory capacity of HFL-1 cells at 72 h (*p* < 0.05, Fig. 3a, b). CM from IgE/Anti IgE stimulated LAD2 mast cells did not have a significantly different effect on fibroblast migration compared to CM from non-stimulated LAD2 cells (Fig. 3a).

Stimulation with CM had no significant effect on fibroblast proliferation, neither in 10% nor 0.4% serum concentrations (Fig. 3c).

### Effect of tryptase on migration and proliferation of fibroblasts

Mast cells contain high concentrations of tryptase in their granules, which they release upon activation. Interestingly, we could observe a time dependent increase in concentrations of tryptase in our co-cultures. As expected, HFL-1 alone did not synthesize any tryptase. There was no significant difference in tryptase release from HFL-1 stimulated with conditioned medium compared to HFL-1 alone (Fig. 4a). To study the effect of tryptase on fibroblast migration, different concentrations of tryptase (10, 50 and 75 ng/mL) were added to the HFL-1 cells [37–39]. The two highest concentrations (50 and 75 ng/mL) of tryptase significantly enhanced the migration after 48 h (*p* < 0.0009) and 72 h (*p* < 0.006), compared to controls (Fig. 4b, c). Tryptase showed a pro-proliferative effect on fibroblasts in medium containing 10% serum at time-point 48 h (*p* < 0.027), but had no significant effect on fibroblast proliferation in 0.4% serum (Fig. 4d). These experiments were also performed with another mast cell protease, chymase, which did not show any effects on either migration or proliferation (data not shown).

### Effect of PAR2 antagonist on fibroblast migration

The PAR2 (F2RL1) gene was differentially expressed in a gene expression analysis, and was highly expressed in HFL-1, but not in LAD2 cells (Fig. 5a). Furthermore, the expression of PAR2 on HFL-1 both in monocultures and in co-culture with LAD2 was verified with immunocytochemistry (Fig. 5b-m). LAD2 cells did not express PAR2 (Fig. 5d). HFL-1 did not express tryptase (Fig. 5g), while LAD2 showed positive staining for tryptase (Fig. 5c). The PAR2 antagonist, P2pal-18S (1 μM and 10 μM), had no significant effect on migration of HFL-1 (Fig. 6a and Additional file 1: Figure S1A). Interestingly, P2pal-18S showed an inhibitory effect (10 μM, *p* < 0.003, 48 h and 72 h) on the pro-migratory effect induced by tryptase, compared to untreated controls (Fig. 6b). This could also be observed in the co-culture system where the enhanced migratory capacity of fibroblasts induced by LAD2 cells was inhibited by P2pal-18S (10 μM, *p* < 0.001, 48 h and 72 h, Fig. 6c; 1 μM, *p* < 0.05, 48 h, Additional file 1: Figure S1B). In contrast, migration of fibroblasts stimulated with conditioned medium was significantly enhanced in the presence of P2pal-18S (10 μM, *p* < 0.005, 48 h; *p* < 0.004, 72 h, Fig. 6d). In summary, LAD2 cells enhanced the migration of HFL-1 by 28% compared to untreated controls. This effect was decreased to 4% by the PAR2 antagonist P2pal-18S (10 μM). Similarly, tryptase enhanced the HFL-1 migration by 21%, while this effect was reduced to 0.015% by P2pal-18S (10 μM) (Fig. 6e).

## Discussion

We have demonstrated for the first time that mast cells (PBdMC and LAD2) and the mast cell mediator tryptase enhance the migratory capacity of human lung fibroblasts (HFL-1). Conditioned medium from LAD2 cells also increased the migration capacity of lung fibroblasts. These findings indicate that factors released by mast cells may trigger and enhance the migration of fibroblasts. This effect may be dependent on cell-cell contact, which in turn could be independent of immunologically activated mast cells. However, our data suggest that tryptase produced and released by mast cells, is one essential soluble factor causing the enhanced fibroblast migration. When inhibiting PAR2 on fibroblasts, we observed an almost complete inhibition of the pro-migratory effect of LAD2 cells and tryptase.

### Migratory and proliferative effects of mast cells on fibroblasts

Several studies have reported that mast cell mediators and especially tryptase may have mitogenic effects on fibroblasts [9, 12]. In the current study, we have showed that PBdMC enhanced the migration capacity of HFL-1 in a co-culture system. Similar results were observed when using the LAD2 mast cell line that induced migration of HFL-1 cells in a concentration dependent manner. The proliferation capacity of fibroblasts was investigated in the same low serum concentration as our migration experiments. As expected, we did not see any effect of mast cells in proliferation experiments with low serum, confirming that we were observing migration and not proliferation in our cell migration assays. However, mast cells significantly decreased fibroblast proliferation in high serum concentration, suggesting that mast cells may have an anti-proliferative effect on fibroblasts, which is serum-dependent. This effect could be a combination of different mast cell proteases acting together [40], higher amounts of protease inhibitors and growth factors or that mast cells may promote the synthesis of anti-fibrotic growth factors, potentially making mast cells protective against remodeling processes in the lung [41]. However, the mechanisms underlying these activities remain unclear**.**

In our experiments, we observed a concentration-dependent increase in fibroblast migration in response to tryptase. In view of published data on mitogenic properties of mast cell tryptase [9, 12], we speculated that the changes in migration could be due to an increased cell proliferation rate. However, we could not see any proliferative effect of tryptase on fibroblasts in the low serum concentration used in our migration experiments. In line with other studies [9, 12], tryptase in high serum concentration significantly increased fibroblast proliferation, suggesting that the interactions between mast cells and fibroblasts are serum-dependent. Thus, our contradictory results on fibroblast proliferation when comparing tryptase and co-cultures with mast cells in higher serum concentration, may be due to cell-cell interaction or other soluble factors released by mast cells.

### Secreted mast cell mediators and cell-cell interactions

In order to study whether the mediators involved in fibroblast-mast cell interactions were released in the cell medium or dependent on cell-cell contact, we investigated the effect of CM from mast cells on fibroblast migration. Interestingly, CM significantly enhanced fibroblast migration, however weaker. When investigating tryptase levels in co-cultures of fibroblasts and mast cells, we observed a time dependent increase in tryptase concentrations, while conditioned medium showed unchanged tryptase concentrations overtime.

In order to investigate whether mast cells release tryptase through an immunological response, mast cells were stimulated with IgE/Anti IgE [42]. However, IgE degranulation of PBdMC attenuated the pro-migratory effect on HFL-1. Importantly, we did not detect any differences between IgE-activated or non-activated LAD2 mast cells or conditioned medium from these cells. However, an explanation to this could be that PBdMC may express different amounts of IgE receptors compared to LAD2 [27]. Another reason for this might be the differences in mediator release between the two degranulation pathways in mast cells; i.e. the anaphylactic degranulation (immunological degranulation) and the piecemeal degranulation (PMD) [43, 44]. It has been suggested that chronic, low-grade partial activation of mast cells could have a role in the pathogenesis of pulmonary fibrosis [45]. Different factors have been proposed to trigger PMD in mast cells in a clinical setting, including chronic psychosocial stress, interactions with regulatory T cells or stimulations of CCL2 and TLR [44]. A selective release of mast cell mediators has been hypothesized to occur during PMD, where the morphology of the granules remains relatively unchanged [46]. However, the pathways behind these actions remain unknown. Dvorak et al., suggested that mast cells may release their granules via PMD in wound healing, and then refill their granule content during this process [44]. These results may explain our findings that IgE-stimulation on mast cells did not further increase fibroblast migration. One important mediator that may be involved in PMD and secreted by fibroblasts is the SCF. SCF is expressed both in the transmembrane and extracellularly by fibroblasts, and is considered to be the driving force in recruiting and activating mast cells towards injured and inflamed sites. SCF binds to c-KIT receptor expressed by mast cells. It is well known that SCF has a crucial role in mast cell development, proliferation and maturation, however several studies have also reported SCF as a mast cell activator inducing degranulation [47–49]. An interesting study reported that fibroblast-SCF interaction with c-KIT receptors on mast cells caused degranulation and activation of mast cells, resulting in synthesis of eotaxin [47, 50]. Eotaxin is an eosinophil-specific chemoattractant, implicated to be dependent on the interactions between fibroblasts and mast cells, however both these cells may produce eotaxin [50]. Hogaboam et al. also showed that the production of SCF by pulmonary human fibroblasts, may be induced by tumor necrosis factor-α, which is produced by mast cells [50, 51]. Wygrecka et al. presented data showing elevated transmembrane SCF expression in fibroblasts from IPF lungs compared to control lungs [5].

However, further experiments are needed in order to clarify the mechanisms causing PMD [52], and to understand the role of mast cells in chronic lung diseases. Interestingly, we noticed some degranulated mast cells without IgE-stimulation using transmission electron microscopy, which could be explained by PMD activation.

In view of our findings and what others have reported about PMD, we propose an important role of cell-cell communication and signaling between mast cells and fibroblasts. We suggest that the interaction between these two cell types could trigger tryptase release through PMD. However, more studies are needed in order to conclude the molecular mechanisms involved in these signaling pathways.

### Role of PAR2 activation on fibroblast migration

In our study we confirmed that HFL-1 expressed mRNA for PAR2, while LAD2 cells did not. Also immunocytochemical stainings confirmed the expression of PAR2s on fibroblasts and the lack of PAR2 on LAD2 cells. A peptide, P2pal-18S, has been reported as a promising antagonist for the PAR2 expressed by human pulmonary fibroblasts [15, 53]. In the present study, the PAR2 antagonist P2pal-18S reduced mast cell-induced fibroblast migration. We could also observe an inhibition of the pro-migratory effect of tryptase, whereas the PAR2 antagonist showed no significant effect on fibroblast migration induced by CM from LAD2 cells. These differences could be due to continuous degranulation of mast cells (PMD) in the co-cultures with mast cells and fibroblast, which did not occur during stimulation with CM alone. In CM there was a limited amount of mast cell components, especially tryptase as we showed in our results. However, in co-cultures mast cells could provide the factors needed for PAR2 activation.

Our results from the migration experiments when investigating the CM from IgE/Anti IgE stimulated mast cells, showed no differences compared to the non-stimulated mast cells. However, this could probably be explained by the limited amount of mast cell tryptase in CM. An explanation to the enhanced fibroblast migration both after stimulation with mast cells and CM could be that different factors are involved, acting through different pathways. In co-cultures, direct cell-cell interactions may be important in addition to soluble factors, including tryptase, present in CM. Cong Lin et al., have demonstrated that PAR2 inhibition by the peptide P2pal-18S reduced pro-fibrotic and pro-inflammatory responses caused by PAR2 activation [15]. Another study has reported that PAR2 activation induced pulmonary fibroblast migration, differentiation and ECM production. This activation occurred by trypsin as a PAR2 agonist, and ERK1/2 activation was suggested as the signaling pathway in fibroblasts [17]. Interestingly, other PAR, such as PAR-4 may instead be involved in mast cell migration [54]. These findings provide a strong indication of mast cell/tryptase/PAR2/fibroblast connection influencing the migration of fibroblasts and may also influence differentiation into myofibroblasts. Previous studies have indicated that fibroblasts from IPF lungs exhibit increased PAR2 expression [19]. High numbers of α-smooth muscle actin (SMA)-expressing myofibroblasts have been observed in IPF and asthma [17, 55] and increased PAR2 activation resulted in increased α-SMA expression [18]. Altogether, these findings support our hypothesis that the mechanism behind the fibroblast migration may occur through the mast cell mediator tryptase and PAR2 activation on fibroblasts.

## Conclusions

In this study, we demonstrated for the first time that human mast cells, the mast cell mediator tryptase and conditioned medium from mast cells enhance the migration capacity of human lung fibroblasts. The promigratory effect of mast cells and mast cell tryptase alone could be inhibited by a PAR2 antagonist, suggesting that these effects are mediated through PAR2 activation. Further studies are warranted to clarify the mechanisms behind these actions that may have a critical role in remodeling processes in the lung.

## Additional file

Additional file 1: **Figure S1.** Effect of different concentrations of PAR2 antagonist on migratory capacity of HFL-1. Two different concentrations of PAR2 antagonist P2pal-18S (1 and 10 μM) were used. Neither of these concentrations had any significant effect on the migratory capacity of the HFL-1 (A). The higher concentration of P2pal-18S (10 μM) inhibited the pro-migratory effect of LAD2 at 48 h and 72 h (A). The lower concentration of of P2pal-18S (1 μM) slightly inhibited the pro-migratory effect of LAD2 at 48 h (B). The migratory capacity of HFL-1 cells was measured at 24, 48 and 72 h as the percentage of cell-occupied space compared to time point 0 h. The statistical analysis was performed using linear mixed models (mean ± SD, *n* = 2 individual experiments for 1 μM and *n* = 3 individual experiments for 10 μM, with 2 technical replicates in each experiment, *p < 0.05, **p < 0.01 and ***p < 0.001). (PPTX 72 kb)

## Abbreviations

aSMA: α-smooth muscle actin
BSA: Bovine serum albumin
CCL2: Chemokine (C-C motif) ligand 2
cDNA: Complementary Deoxyribonucleic acid
CM: Conditioned medium
COPD: Chronic obstructive pulmonary disease
DMEM: Dulbecco’s modified eagle medium
ECM: Extracellular matrix
ELISA: Enzyme-linked immunosorbent assay
ERK1/2: Extracellular signal–regulated kinases 1/2
FCIII: Fetal clone serum 3
HFL-1: Human fetal lung fibroblasts
IgE: Immunoglobulin E
IL-3: Interleukin 3
IL-6: Interleukin 6
IPF: Idiopathic pulmonary fibrosis
PAR2: Protease activated receptor 2
PBdMC: Human peripheral blood derived mast cells
PBS: Phosphate-Buffered Saline
PCR: Polymerase chain reaction
PEST: Penicillin-Streptomycin
PMD: Piecemeal degranulation
SCF: Stem cell factor
SEM: Scanning electron microscopy
TBS: Tris buffered saline
TEM: Transmission electron microscopy
