# Supplementary figures and images for: Mast cells and mast cell tryptase enhance migration of human lung fibroblasts through protease-activated receptor 2
Source: Cell Commun Signal. 2018 Sep 15;16:59. doi: 10.1186/s12964-018-0269-3 (PMC6139170; doi:10.1186/s12964-018-0269-3)

## Slide 1
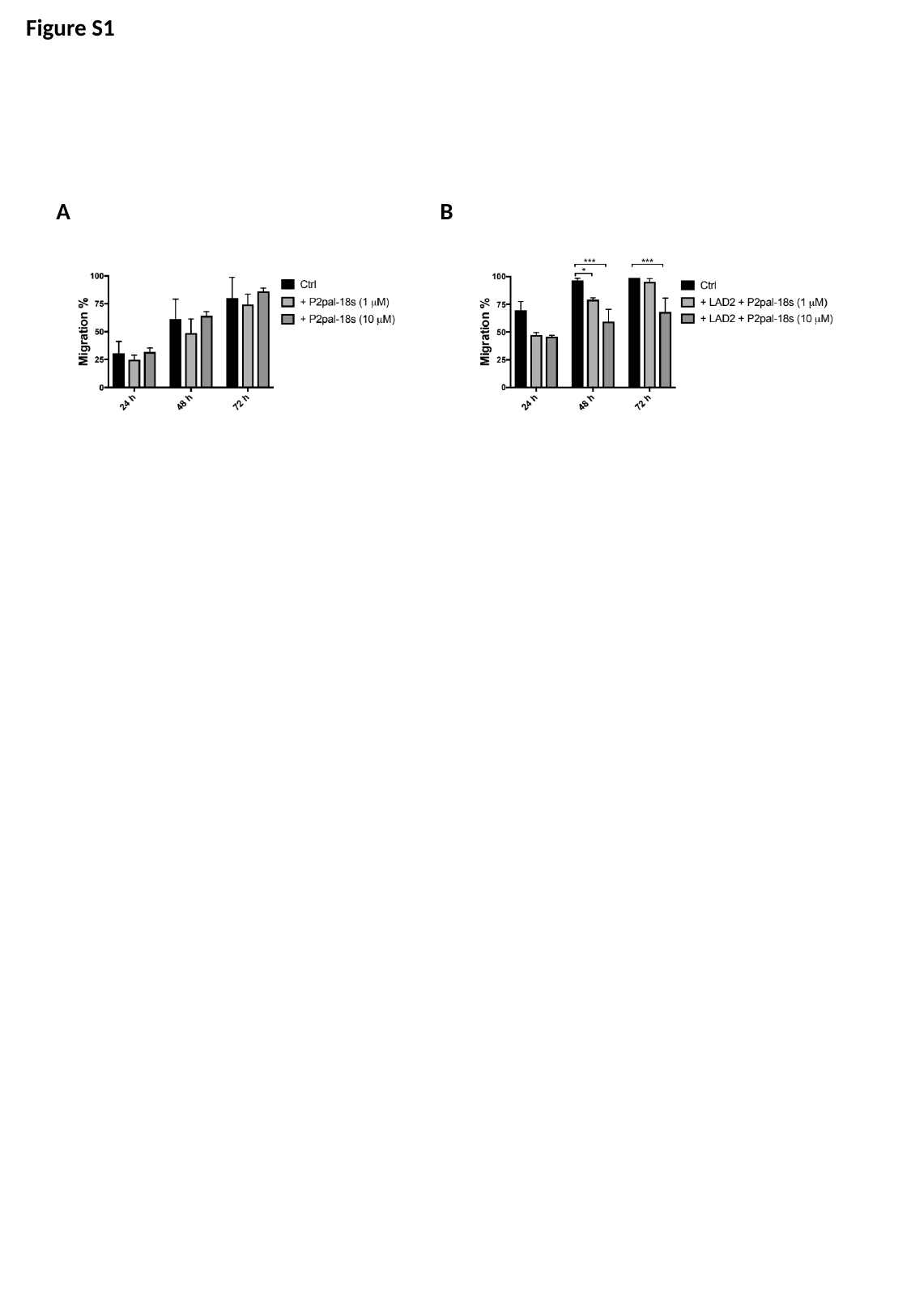

Supplement: Supplementary file 1 — Figure S1. Effect of different concentrations of PAR2 antagonist on migratory capacity of HFL-1. Two different concentrations of PAR2 antagonist P2pal-18S (1 and 10 μM) were used. Neither of these concentrations had any significant effect on the migratory capacity of the HFL-1 (A). The higher concentration of P2pal-18S (10 μM) inhibited the pro-migratory effect of LAD2 at 48 h and 72 h (A). The lower concentration of of P2pal-18S (1 μM) slightly inhibited the pro-migratory effect of LAD2 at 48 h (B). The migratory capacity of HFL-1 cells was measured at 24, 48 and 72 h as the percentage of cell-occupied space compared to time point 0 h. The statistical analysis was performed using linear mixed models (mean ± SD, n = 2 individual experiments for 1 μM and n = 3 individual experiments for 10 μM, with 2 technical replicates in each experiment, *p < 0.05, **p < 0.01 and ***p < 0.001). (PPTX 72 kb) [file 12964_2018_269_MOESM1_ESM.pptx]
